# Associations of Elevated Red Cell Distribution Width (RDW) with Decreased Physical and Cognitive Function in Older Adults, and The Potential Mediation by Mitochondrial Energetics: The Study of Muscle, Mobility and Aging (SOMMA)

**DOI:** 10.14336/AD.2024.1724

**Published:** 2026-04-22

**Authors:** Kyoung Min Kim, Li-Yung Lui, Theresa Mau, Paul M. Coen, Steven R Cummings

**Affiliations:** ^1^Division of Endocrinology, Department of Internal Medicine, Yongin Severance Hospital, Yonsei University College of Medicine, Yongin, South Korea.; ^2^San Francisco Coordinating Center, California Pacific Medical Center Research Institute, San Francisco, California, USA.; ^3^Department of Epidemiology and Biostatistics, University of California, San Francisco, California, USA.; ^4^AdventHealth, Translational Research Institute, Orlando, Florida, USA.

**Keywords:** Red cell distribution width, Mitochondria, Aging, Biomarker

## Abstract

We analyzed the association between RDW and skeletal muscle mitochondrial energetics and how skeletal muscle mitochondrial energetics may mediate the associations of RDW with physical and cognitive performance. The study analyzed cross-sectional baseline data from the Study of Muscle, Mobility and Aging (SOMMA) that enrolled 864 participants aged 70 and older (mean=76.3 years). RDW, clinical and demographic parameters were assessed. Comprehensive evaluations were conducted for both physical and cognitive function using objective and subjective measures. Elevated RDW values were significantly correlated with decreased physical performance, evidenced by reduced cardiorespiratory fitness (VO_2peak_) and longer time to 400 m Walk, alongside impaired cognitive performance. Higher RDW values also demonstrated robust negative associations with various measurements of mitochondrial energetics, including maximal ATP production and oxidative phosphorylation. Mediation analysis revealed that impaired mitochondrial function partly mediated the associations between RDW values and VO_2peak_, and other physical and cognitive performance. These findings suggest that higher RDW is associated with declines in various physical and cognitive performance, with skeletal muscle mitochondrial energetics serving as a potential mediating factor. Causal inferences about potential mediation are limited by the cross-sectional design of the study. Nevertheless, the findings highlight the value of RDW as a potential biomarker for age-related declines in physical and cognitive function partly mediated by mitochondrial energetics.

## INTRODUCTION

Red cell distribution width (RDW) is a component of complete blood cell count (CBC) that measures the variation in red blood cell (RBC) size [1,2]. Traditionally, it has been used in diagnosing and differentiating types of anemia in the hematologic field. However, in addition to its diagnostic roles in anemia, RDW has gained attention for its relationships with various other non-hematologic chronic diseases related to aging: a higher RDW value is associated with an increased risk of cardiovascular disease [3,4], renal insufficiency, dementia, risk of falls, and fracture [5,6]. Higher RDW values have also correlated with impairment in physical function, progression of functional deterioration and greater mortality in ambulatory community populations as well as hospitalized patients [7,8]. Although the causality remains unclear, it has been argued that elevated RDW could be a potential biomarker for accelerated aging processes and related phenotypes.

Aging is associated with a myriad of physiological changes, and the decline in mitochondrial function is one of the pivotal aspects of aging [9]. Mitochondrial energetics, encompassing oxygen consumption, adenosine triphosphate (ATP) synthesis, and maintenance of cellular redox, undergoes significant deterioration with aging [10]. The age-related decrease in mitochondrial energetics at the cellular level is known to be closely linked to various phenotypic changes in human aging [11].

Although red blood cells inherently lack mitochondria, elevated RDW values may indirectly reflect broader systemic disturbances, such as chronic inflammation and oxidative stress, which negatively affect mitochondrial function for various organs including skeletal muscle. Additionally, the production of red blood cells by hematopoiesis and the clearance of senescent and damaged red blood cells by the reticuloendothelial system require energy from mitochondria. Decrease in mitochondrial energetics might impair these functions and increase the variability in the size of circulating cells [12,13]. Given the established importance of mitochondrial energetics in sustaining physical and cognitive function, we hypothesize that mitochondrial dysfunction could partially mediate the associations between elevated RDW and age-related functional impairments. To investigate this, we performed mediation analyses evaluating mitochondrial energetics as a potential mechanistic link connecting higher RDW values to age-related declines in functional capacities.

The present study utilizes cross-sectional data from the Study of Muscle, Mobility and Aging (SOMMA), a well-characterized cohort specifically designed to investigate cellular and physiological aspects of aging in older adults. SOMMA includes detailed assessments of physical and cognitive performance, as well as mitochondrial energetics from 31P magnetic resonance spectroscopy of the quadriceps and mitochondrial respirometry from biopsies of the vastus lateralis. These assessments in SOMMA provided an ideal dataset to explore associations between RDW, mitochondrial dysfunction, and age-related functional decline.

## MATERIALS AND METHODS

### Study subjects in SOMMA Study

Participants aged 70 and older were recruited for the Study of Muscle, Mobility and Aging (SOMMA) (https://www.sommaonline.ucsf.edu/) from April 2019 to December 2021 across 2 clinical sites; the University of Pittsburgh and Wake Forest University School of Medicine. Briefly, participants were eligible for this study if willing and able to complete a muscle tissue biopsy and magnetic resonance (MR) [14]. The exclusion criteria included the following:

Inability to walk 400 meters or climb a flight of stairs; gait speed ≤ 0.6 m/s; BMI ≥ 40 kg/m^2^; an active malignancy or advanced chronic disease (e.g., severe heart or lung disease, severe kidney disease on dialysis, Parkinson’s disease, dementia) that would prevent completing baseline assessments; limit their likelihood of providing follow-up data; contraindication to a muscle biopsy; or the presence of magnetic resonance (MR) incompatible implants. In-person assessments ensured that all were able to walk 400 m at enrollment [15]. All participants provided written informed consent, and the study was approved by the Western IRB-Copernicus Group (WCG) Institutional Review Board (WCGIRB #20180764).

### Assessments

#### Demographic and clinical parameters

Baseline assessments for SOMMA were collected over three visits and included questionnaires on demographic information including age based on self-reported date of birth, self-reported sex, ethnicity, and race. Height, weight and waist circumference were measured, and body mass index (BMI) was calculated as weight divided by square of height (kg/m^2^). Presence of diabetes was identified by self-report, use of prescribed hypoglycemic medication, or having an HbA1c 6.5%. Blood pressure and heart rates were recorded following a 5-minute rest period.

#### CBC assays

Among the 879 participants who attended the baseline assessment, 864 participants had blood samples sufficient for CBC analysis. CBC were measured by a hematology analyzer as standard methods at Local Quest Diagnostic Laboratory in Pittsburgh and Wake Forest Medical Center laboratory. Among routine CBC parameters, the RDW is assessed by calculation of variations in RBC size, as measured by mean corpuscular volume (MCV). The values of RDW (%) are calculated using the following formula: RDW (%) = (Mean Corpuscular Volume/Standard Deviation of MCV) × 100. The reference ranges for RDW values were set at 11.0-15.4%.

#### Skeletal muscle maximal mitochondrial energetics

To assess ex vivo skeletal muscle mitochondrial energetics, percutaneous biopsies of the musculus vastus lateralis were obtained under local anesthesia and controlled prior exercise. [16,17] Approximately 2-3mg of myofiber bundles was used for chemical permeabilization with saponin for high-resolution respirometry of permeabilized muscle fiber (PMF) bundles with Oxygraph 2K instruments (Oroboros Inc., Innsbruck, Austria). Maximal complex I- and II-supported oxidative phosphorylation (Max OXPHOS_CI+CII_) was measured by sequentially adding substrates: adenosine diphosphate (ADP, incrementally up to 4.2 mM), pyruvate (10 mM), glutamate (10 mM), and succinate (10 mM). Maximal uncoupled respiration was also measured. Additionally, maximal mitochondrial ATP production (ATP_max_) was assessed in quadriceps muscle via ^31^P MRS using a 3-Tesla MR magnet (Siemens Prisma or Skyra) by measuring phosphocreatine recovery kinetics after isometric leg extension exercises [16].

#### Cardiopulmonary exercise testing (CPET) and Borg rating of perceived exertion

Participants completed a modified Balke cardio-pulmonary exercise test (CPET) to assess peak oxygen consumption (VO_2peak_). The CPET started with a 5-minute warm-up walk, followed by increasing treadmill incline by 2.5% every 2 minutes to a maximum of 10%, and then speed increments of 0.5 mph every 2 minutes until fatigue. The Borg rating of perceived exertion scale was used to assess exercise intensity [18,19].

#### Short Physical Performance battery (SPPB)

The 12-point SPPB assessed lower extremity function through gait speed, standing balance, and chair stands [20]. Each component was scored from 0 to 4. Gait speed was measured over a 4-meter course, while the chair stand test quantified the time to complete five consecutive stands without arm support. The total SPPB score, ranging from 0 to 12, reflected overall functional performance, with higher scores indicating better function [21,22].

#### Usual-Paced 400-meter Long Distance Corridor Walk

In the 400-meter walk test, participants were directed to complete 10 laps around a predetermined course at their usual pace, ensuring they did not exert themselves excessively. They were permitted to pause and stand still for a maximum of one minute if needed during the test [23].

#### Lower leg extension muscle power and grip strength

Knee extensor leg power was assessed using a Keiser pneumatic resistance device. The right leg was primarily used, barring previous joint replacement or motion limitations. Resistance started at 40 pounds and increased to the participant’s maximum capacity. Power output at 40%, 50%, 60%, and 70% of 1-RM was recorded, with the highest value standardized to body weight (Watts/kg) [24]. Grip strength was assessed using a Jamar hand dynamometer, with participants performing one practice and two maximal-effort trials per hand. The highest force (kg) was recorded as maximum grip strength [25].

#### Cognitive performance (Digit Symbol Coding Test & Trail-making Task B)

The Digit Symbol Coding Test (DSC) and the Trail-making Task B (Trails B) were conducted to comprehensively evaluate cognitive performance. The DSCT required participants to match symbols with corresponding digits using a provided key, completing as many accurate pairings as possible within 120 seconds. Scores ranged from 0 to 133, with higher scores indicating better cognitive function [26]. Trails B evaluated executive function, processing speed, and visuospatial ability by having participants sequentially connect numbers and letters in alternating numerical and alphabetical order. Performance was measured by the total time (seconds) required to complete the task, reflecting cognitive agility [27].

#### Objectively measured physical activity

Activity levels were assessed using two devices: the activPAL, attached to the thigh to track daily step count, and the ActiGraph GT9x, worn on the wrist. Participants wore these devices for seven consecutive days starting from their initial visit ("Day 1"). Data were included if the devices were worn for at least 17 hours per day. Total daily activity counts were analyzed. Among the variables collected, total daily activity counts were included in the analyses [28].

### Statistical analysis

Data were presented as mean ± standard deviation for continuous values, or as numbers (percentages) for categorical values. RDW values were categorized into six groups:<12, 12-12.4, 12.5-12.9, 13.0-13.4, 13.5-14.4 and ≥14.5%. Participant characteristics were compared across the RDW categories using ANOVA and Chi-square test. Before parametric tests, we confirmed the normal distribution of variables using the Shapiro-Wilk test. Non-normally distributed variables were analyzed using corresponding non-parametric tests, including the Kruskal-Wallis test. The associations between RDW and parameters related to mitochondrial energetics were analyzed by Pearson correlation coefficient. To assess linear trends across RDW categories, the Cochran-Armitage test for trends in proportions and Cuzick’s test for trends in mean values were employed. Covariates included in the regression analyses were age, hemoglobin levels, and diabetes status. Each covariate was selected based on biological plausibility and evidence from existing literature: age was adjusted as it is strongly associated with RDW, mitochondrial energetics, and age-related declines in physical and cognitive functions. Hemoglobin levels were included given their direct relationship with RDW. Diabetes status was incorporated due to its established relationship with impaired mitochondrial energetics and physical and cognitive dysfunction in older populations.

**Figure 1.**
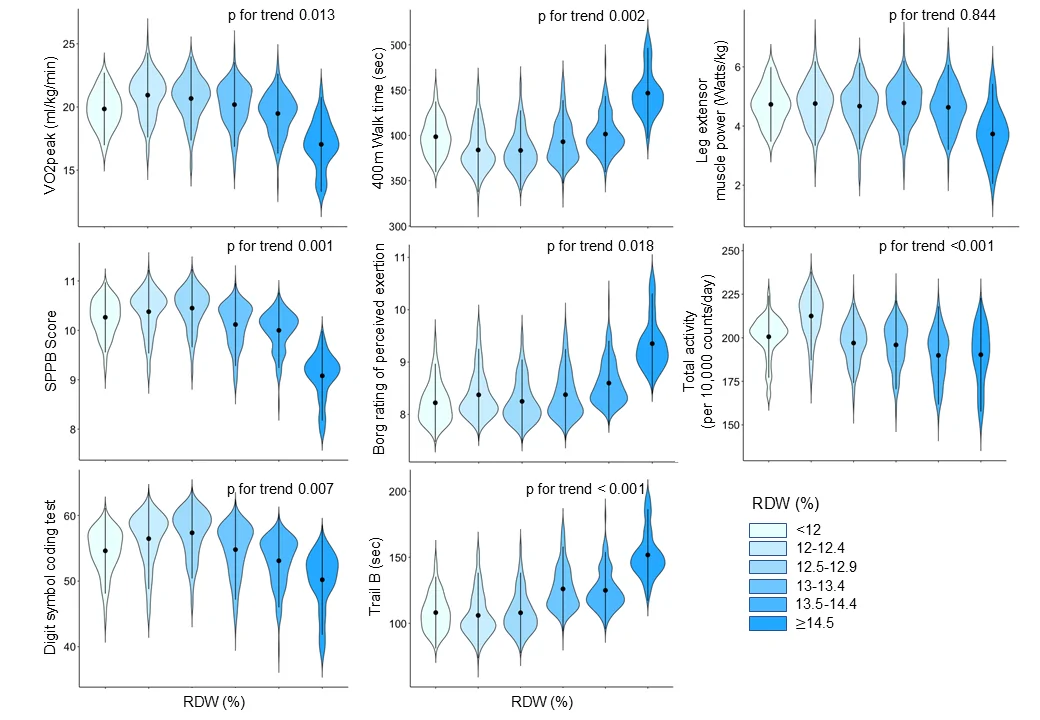
**The values of physical and cognitive assessments among each RDW category**. Higher RDW values were associated with poorer physical and cognitive performance. Data are presented as violin plots with adjusted mean + SD with covariates of age and hemoglobin. Sample sizes for each RDW category are as follows: RDW <12% (n=53), 12%-12.4% (n=167), 12.5-12.9%(n=258), 13%-13.4%(n=204), 13.5%-14.4%(n=138), ≥14.5(n=44). P for trend was calculated using a generalized linear model (GLM) with RDW groups as an ordinal variable. SPPB, Short Physical Performance battery; RDW, red cell distribution width; SEM, standard error of mean.

All these selected covariates also provided significant associations for RDW values in univariate regression analyses. As skeletal muscle mitochondrial energetics differ between men and women, we conducted interaction analyses between RDW and sex. In addition, since several types of anemia increases RDW value, interaction terms between RDW categories and anemia status (defined as hemoglobin <13.0 g/dL for men and <12.0 g/dL for women, based on WHO criteria) were also tested using linear regression to examine potential effect modifications. Mediation analyses were conducted to evaluate the role of mitochondrial energetics (assessed by ATPmax) as a potential mediator in the relationship between RDW and functional outcomes. These analyses were performed using the 'lavaan' package in R. The mediation model tested the indirect (mediated) and direct associations between RDW (independent variable), mitochondrial energetics (mediator), and physical and cognitive functional outcomes. The key assumptions underlying the mediation analyses, including linearity of relationships among variables, absence of multicollinearity, and normal distribution of residuals, were verified prior to modeling. The robustness of mediation effects was evaluated using a bootstrap approach with 5000 resampling iterations to estimate accurate confidence intervals for indirect effects, minimizing dependency on parametric assumptions. The proportion mediated was calculated by dividing the indirect effect by the total effect and was presented as a percentage. Statistical significance was set at a two-tailed p-value <0.05. All analyses were performed using R statistical software (version 4.2.2; R Project for Statistical Computing).

## RESULTS

A total of 864 participants with RDW values were included in the current analysis. The mean age was 76.3 (± 5.0) years, with 58.8% being, and 17.9% having diabetes mellitus. RDW values range from 11.3 to 19.7% and were highly right-skewed with a median value of 12.9%. In the associations with baseline clinical parameters, RDW values were positively associated with weight, waist circumference and BMI, indicating subjects with greater variability of RBC size were more likely to be obese. Subjects having higher RDW are more likely to have diabetes mellitus. In contrast, it provided negative associations with other RBC-related parameters including hemoglobin, hematocrit, and mean corpuscular hemoglobin (MCH) (p < 0.05, respectively) (Table 1).

**Table 1.** Baseline characteristics of study subjects in total and among RDW categories, median [IQR] or N (%).

	Total Subjects	RDW (%)						p value*	P for Trend
		<12	12-12.4	12.5-12.9	13-13.4	13.5-14.4	≥14.5		
**N**	864	53	167	258	204	138	44		
**Female (%)**	508 (58.8)	38 (71.7)	103 (61.7)	150 (58.1)	107 (52.5)	79 (57.2)	31 (70.5)	0.068	0.388
**Race**								<0.001	<0.001
**White**	737 (85.3)	51 (96.2)	149 (89.2)	240 (93.0)	173 (84.8)	101 (73.2)	23 (52.3)		
**Others**	127 (14.7)	2 (3.8)	18 (10.8)	18 (7.0)	31 (15.2)	37 (26.8)	21 (47.7)		
**Age, years**	75.0 [72.0,79.0]	75.0 [73.0,78.0]	75.0 [72.0,79.0]	75.0 [72.0,79.0]	75.0 [73.0,80.0]	75.0 [72.0,79.0]	75.5 [73.0,79.0]	0.659	0.528
**Height, m**	1.65 [1.58,1.73]	1.63 [1.58, 1.70]	1.65 [1.58, 1.73]	1.65 [1.58, 1.73]	1.67 [1.60, 1.73]	1.65 [1.59, 1.74]	1.62 [1.56, 1.72]	0.349	0.231
**Weight, kg**	75.2 [65.2,85.2]	72.5 [61.0,77.5]	74.0 [63.0,80.1]	74.0 [63.6,85.4]	76.7 [68.0,85.4]	80.4 [68.9,89.4]	76.0 [64.2,89.4]	<0.001	<0.001
**BMI, kg/m^2^**	27.0 [24.3,30.7]	26.2 [23.8,29.3]	26.0 [23.7,28.4]	27.0 [23.8,30.2]	27.4 [24.5,31.0]	29.2 [25.6,32.6]	28.6 [24.7,32.5]	<0.001	<0.001
**DM (%)**	155 (17.9)	7 (13.2)	15 (9.0)	31 (12.0)	54 (26.5)	33 (23.9)	15 (34.1)	<0.001	<0.001
**WC, cm**	94.0 [84.4,103.2]	90.5 [79.8,98.2]	90.2 [82.0,99.2]	94.0 [83.2,103.7]	96.4 [86.5,104.2]	97.2 [88.1,105.0]	94.7 [85.1,106.6]	<0.001	<0.001
**SBP, mmHg**	129.0 [119.0,141.0]	126.5 [114.0,139.5]	130.0 [118.8,141.8]	129.5 [118.0,140.0]	127.8 [119.0,141.0]	130.8 [121.0,142.0]	132.0 [118.0,144.5]	0.314	0.189
**DBP, mmHg**	69.5 [63.5,77.5]	71.0 [65.0,76.5]	71.0 [65.5,77.2]	68.5 [63.0,77.5]	69.0 [62.8,76.5]	70.5 [63.5,78.5]	69.5 [63.8,79.8]	0.402	0.999
**PR,/min**	67.5 [61.0,74.8]	65.5 [60.5,71.5]	66.5 [59.8,73.5]	68.2 [61.5,74.5]	66.8 [60.5,74.0]	69.0 [62.0,76.5]	67.8 [62.8,79.0]	0.281	0.031
**WBC, x10^3/μL**	5.9 [5.0,7.0]	5.8 [4.7, 6.9]	5.9 [5.0, 6.8]	5.9 [5.0, 6.9]	5.9 [5.0, 7.1]	5.8 [4.9, 7.0]	6.0 [5.0, 7.0]	0.766	0.217
**Hematocrit, %**	40.4 [38.3,42.7]	40.4 [38.1,42.0]	40.7 [38.5,42.5]	40.9 [38.7,43.1]	40.5 [38.6,43.0]	39.9 [37.6,42.4]	38.8 [35.3,41.3]	0.005	0.013
**Hemoglobin, g/dL**	13.6 [12.8,14.5]	13.9 [13.0,14.5]	13.7 [12.9,14.5]	13.8 [13.0,14.6]	13.6 [12.8,14.5]	13.4 [12.5,14.3]	12.7 [11.3,13.2]	<0.001	<0.001
**MCH, pg**	30.7 [29.6,31.8]	31.9 [31.2,32.9]	31.4 [30.5,32.3]	30.8 [30.0,31.8]	30.4 [29.4,31.3]	29.8 [28.6,30.7]	28.6 [25.9,29.7]	<0.001	<0.001
**Platelets, x10^3/μL**	229.0 [193.0,265.0]	222.0 [182.0,258.0]	232.0 [197.5,263.0]	227.0 [191.0,264.0]	226.0 [188.5,264.5]	233.5 [200.0,275.0]	249.5 [206.5,286.5]	0.281	0.094

Data are presented as median [IQR] for continuous variables, and as frequencies (percentages) for categorical variables.

* P-values were calculated using the Kruskal-Wallis test for continuous variables and the Chi-square test for categorical variables.

P for trend was calculated using a generalized linear model (GLM) with RDW groups as an ordinal variable.

BMI, body mass index; DM, diabetes mellitus; WC, waist circumstance; SBP, systolic blood pressure: DBP, diastolic blood pressure; PR, purse rate;

WBC, white blood cell count; MCH, mean corpuscular hemoglobin; IQR, inter quartile range;

### Associations between RDW value and physical and cognitive functions

Higher RDW values, which means greater variability in RBC size, are significantly associated with reduced or impaired physical functions in many of the objective and subjective assessments measured except grip strength, and all these associations except leg muscle power remained significant even after adjusting age or further adjusting hemoglobin levels. (adjusted *p* for trends <0.05 for all, adjusted p for trends 0.844 for leg muscle power) (Fig. 1): The higher RDW categories, the lower VO_2_peak, longer 400m walk time and lower SPPB scores. Borg rating of perceived exertion was also greater and total physical activities were lower in older adults with higher RDW categories. Cognitive function parameters, including attention and concentration, exhibited significant trends correlating with RDW levels, demonstrating a decrease in DSC performance and an increase in Trails B scores as RDW categories increased. Further adjusting for diabetes had no meaningful effect on those associations. There were no interactions with sex or anemia in these all-observed associations (Data not shown). Additionally, when we defined individuals exhibiting concurrent impairments in both cognitive and physical function—based on poor performance in both the Trail-making Task B (≥ 180 secs) and SPPB (≤ 9 score)—the subgroup with dual impairments demonstrated significantly higher RDW values compared to those exhibiting impairment in either cognitive or physical function alone (Supplementary Table 1).

### Associations between RDW values and mitochondrial energetics

**Figure 2.**
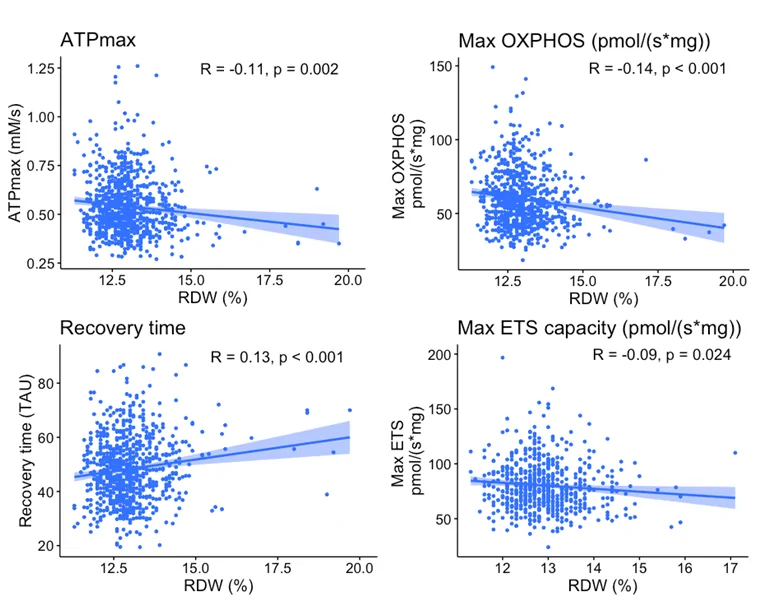
**Associations between RDW values and mitochondrial energetics-related parameters (Total n = 864)**. Higher RDW values were significantly associated with impaired mitochondrial energetics, as indicated by lower ATP_max_, Max OXPHOS, Max ETS and lower recovery time. Data are presented as Pearson correlation coefficients, with corresponding R and p values. ATP_max_ – maximal production of adenosine triphosphate; Max OXPHOS – maximal oxidative phosphorylation; Max ETS – uncoupled, maximal electron transport system capacity.

We also evaluated the correlations between RDW and various measures of muscle mitochondrial energetics by ^31^P MR spectroscopy (ATP_max_) and by respirometry in biopsies of the vastus lateralis. Notably, RDW values demonstrated robust associations with all assessed mitochondrial parameters. Specifically, there was a discernible negative correlation between RDW and ATP_max_, Max OXPHOS, and Max ETS capacity. Conversely, a positive association was observed between RDW values and the PCr recovery time (Fig. 2). These associations remained statistically significant after adjusting for age, but lost statistical significance after further adjusting for hemoglobin in ATP_max_ and Max ETS capacity (data not shown). There were also no interactions with sex (Data not shown).

**Table 2.** Mediation analyses of mitochondrial energetics in the associations between RDW and physical, or cognitive function.

	Mediation analysis						
	ADE		ACME		Total effect		
	Direct	p-value	Indirect	p-value	Total	p-value	Proportion mediated
**peakVO_2_ (ml/kg/min)**	-0.35	<0.001	-0.20	0.004	-0.55	<0.001	36%
**400 m Walk (time)**	4.29	0.012	2.30	0.008	6.59	<0.001	35%
**SPPB**	-0.16	<0.001	-0.04	<0.001	-0.19	<0.001	18%
**Borg Rating of Perceived Exertion**	0.12	0.028	0.04	<0.001	0.16	<0.001	25%
**Total activity F(per 10,000 counts/day)**	-4.54	0.008	-0.39	0.048	-4.93	<0.001	7%
**Digit Symbol Coding Test (number of correct)**	-0.96	0.020	-0.26	<0.001	-1.23	0.008	21%
**Trails B (Total time)**	8.24	<0.001	1.16	0.012	9.41	<0.001	12%

The table presents the ADE, representing the direct association between RDW and functional outcomes, and the ACME, indicating the indirect effect mediated by mitochondrial energetics. The total effect is the sum of ADE and ACME, while the proportion mediated represents the percentage of the total effect explained by mitochondrial energetics.

### Mitochondrial energetics partially mediates the associations between RDW and functional parameters

Our study investigated the potential mediating role of impaired mitochondrial energetics, as evidenced by reduced ATP_max_ levels in the observed significant associations between elevated RDW values and diverse physical and cognitive performance outcomes. We focused ATP_max_ because the association between RDW and ATP_max_ was stronger than for other measurements of mitochondrial energetics. Mediatization revealed that mitochondrial energetics significantly influenced all these observed associations to varying degrees. Specifically, the relationship between VO_2_peak and RDW values was 36% mediated by decreased mitochondrial energetics, 400 m Walk by 35%, Borg Rating of Perceived Exertion by 25% and the DSCT outcomes by 21% (Table 2 and Supplementary Fig 1). The associations were not changed by adjusting for age, hemoglobin levels and diabetes (data not shown).

## DISCUSSION

The present study investigated the associations of RDW values with age-related alterations in physical and cognitive performance, while also determining if these relationships were mediated by muscle mitochondrial energetics. The results demonstrated notable associations between elevated RDW values and reduced physical and cognitive abilities in a cohort of older adults from the Study of Muscle, Mobility and Aging (SOMMA). Many of these associations were partially mediated by poorer mitochondrial energetics.

Our study findings substantiate that RDW values, indicative of increased heterogeneity in RBC size, correlate with diminished physical functioning. This correlation is apparent through objective measures, including cardiopulmonary fitness, leg muscle power, and 400m walk time, as well as through subjective evaluations. These results align with prior research demonstrating a link between heightened RDW levels and reduced physical capabilities in the older adult population [7]. Moreover, the study extends these observations by identifying significant associations between RDW values and cognitive performance, corroborating earlier investigations that have explored the relationship between RDW levels and cognitive decline, including dementia [7,29].

Although the mechanisms underlying the increase in RDW values with aging and age-related functional declines have not been fully elucidated, several hypotheses have been proposed. Clonal Hematopoiesis (CH), characterized by blood cell proliferation from a single hematopoietic stem cell clone, results from age-related mutations, and the presence of CH is associated with heightened risks of chronic diseases such as diabetes and cardiovascular disease [30-34]. It is believed that CH is one of the major factors that lead to an increase of variability in RBC size, resulting in greater RDW values [32,34]. Cellular aging may also alter cell sizes, including that of RBCs, due to the deteriorative changes in the extracellular membrane and cytoskeleton [35-37]. Chronic inflammation or nutritional deficiencies associated with the aging process may also contribute to the increased variability in RBC size. Despite the uncertainty surrounding the causal relationship between increased variability of RBC size and age-related phenomena, elevated RDW values may still be a marker of accelerated aging and its associated manifestations.

Mitochondrial energetics, the cornerstone of cellular metabolism, also undergoes significant alterations with aging, manifesting in diminished efficiency and capacity of energy production [11]. This decline is characterized by a reduction in maximal ATP production, impaired oxidative phosphorylation, and decreased electron transport ETS capacity [9,10]. Our study investigated the relationship between RDW values and mitochondrial energetics, and we found the robust associations between RDW values and various indicators of mitochondrial function. These findings imply that high RDW levels are not only associated with declines in various functional deteriorations with aging but may also be an indicator of impaired mitochondrial energetics. Indeed, despite the absence of mitochondrial in red blood cells, elevated RDW values may indirectly represent accelerated aging processes driven by underlying mechanisms such as impaired hematopoiesis and clearance of senescent red blood cells and chronic inflammation and oxidative stress that may impair mitochondrial function across multiple tissues [10,12,13]. In skeletal muscle and neural tissues specifically, this mitochondrial dysfunction could manifest as impaired physical and cognitive functions. The mediation analyses further supported the relevance of mitochondrial energetics as a partial mechanistic link connecting elevated RDW values with age-related functional declines demonstrating that mitochondrial dysfunction mediated 35% of the association with physical performance assessed by 400 m Walk test, 36% for cardiorespiratory fitness as measured by maximum oxygen consumption (VO_2_peak), and 21% for DSCT measures. Thus, elevated RDW could be valuable as a simple and accessible biomarker, reflecting systemic physiological dysregulation associated with mitochondrial impairment and functional deterioration in aging.

In the present study, we observed no significant interactions between anemia status and RDW in their associations with physical and cognitive performance measures, and these associations remained consistent after adjustment for hemoglobin levels. Although anemia itself can elevate RDW values and independently influence tissue function, our current findings, consistent with prior research, indicate that associations between RDW and functional impairments persist independently of anemia status. These results further support the utility of RDW as a reliable biomarker of aging-related physiological decline, irrespective of anemia.

Clinically, our findings suggest that RDW measurement, an easily obtainable and cost-effective laboratory parameter, could serve as an early indicator for identifying older individuals at increased risk of age-related physical and cognitive impairments. Given the widespread availability and affordability of RDW testing, incorporating it into standard geriatric assessments may enhance early risk stratification in older populations. However, further research is needed to determine whether monitoring RDW values in geriatric clinical practice could facilitate timely intervention strategies—such as lifestyle modifications, nutritional support, or anti-inflammatory therapies—to potentially mitigate functional decline.

The strengths of our study include a comprehensive assessment of physical and cognitive functions with standardized protocols, as well as gold standard measures of skeletal muscle mitochondrial energetics in a well-phenotyped group of older adults. However, there are several limitations that should be acknowledged. First, our study employed a cross-sectional design, which inherently restricts causal interpretation; thus, the observed associations between RDW values, mitochondrial dysfunction, and functional impairments should be interpreted cautiously. We emphasize that elevated RDW values themselves do not directly cause declines in mitochondrial function or physical and cognitive performance. Instead, we propose that RDW may serve as an indirect marker reflecting impaired hematopoiesis and clearance of senescent red blood cells or physiological disturbances, such as inflammation. Future longitudinal studies are essential to clarify these proposed mechanistic pathways and establish causal directions more conclusively. Secondly, the predominantly Caucasian and older age of the SOMMA cohort limits generalizing our results to younger age groups or more ethnically diverse populations. Lastly, despite adjusting for hemoglobin and diabetes status, other unmeasured confounders such as nutritional status or chronic inflammatory markers may have influenced both RDW and mitochondrial function, potentially affecting the observed associations. Additional research incorporating these factors is necessary to comprehensively clarify and validate the findings presented here.

In conclusion, our study provides evidence for the associations between RDW values and diverse physical and cognitive functions and highlights the potential mediating role of impaired muscle mitochondrial energetics in older adults. This research adds to the expanding body of evidence regarding the clinical relevance of RDW values as an aging biomarker.

## Supplementary Materials

The Supplementary data can be found online at: www.aginganddisease.org/EN/10.14336/AD.2024.1724.

## Data Availability

All SOMMA data are publicly available via a web portal. Updated datasets are released approximately every 6 months (https://sommaonline.ucsf.edu/).
