# Supplementary material for: Associations of Elevated Red Cell Distribution Width (RDW) with Decreased Physical and Cognitive Function in Older Adults, and The Potential Mediation by Mitochondrial Energetics: The Study of Muscle, Mobility and Aging (SOMMA)
Source: Aging Dis. 2025 Apr 22;17(3):1654–63. doi: 10.14336/AD.2024.1724 (PMC13061556; doi:10.14336/AD.2024.1724)
Supplement: Supplementary file 1 — The Supplementary data can be found online at: www.aginganddisease.org/EN/10.14336/AD.2024.1724. [file AD-17-3-1654-s.pdf]

SUPPLEMENTARY DATA

Associations of Elevated Red Cell Distribution Width (RDW) with Decreased Physical and Cognitive Function in Older Adults, and The Potential Mediation by Mitochondrial Energetics: The Study of Muscle, Mobility and Aging (SOMMA)

Kyoung Min Kim, Li-Yung Lui, Theresa Mau, Paul M. Coen, Steven R Cummings

© 2025. Kim KM et al. Published online at http://www.aginganddisease.org/EN/10.14336/AD.2024.1724

SUPPLEMENTARY DATA

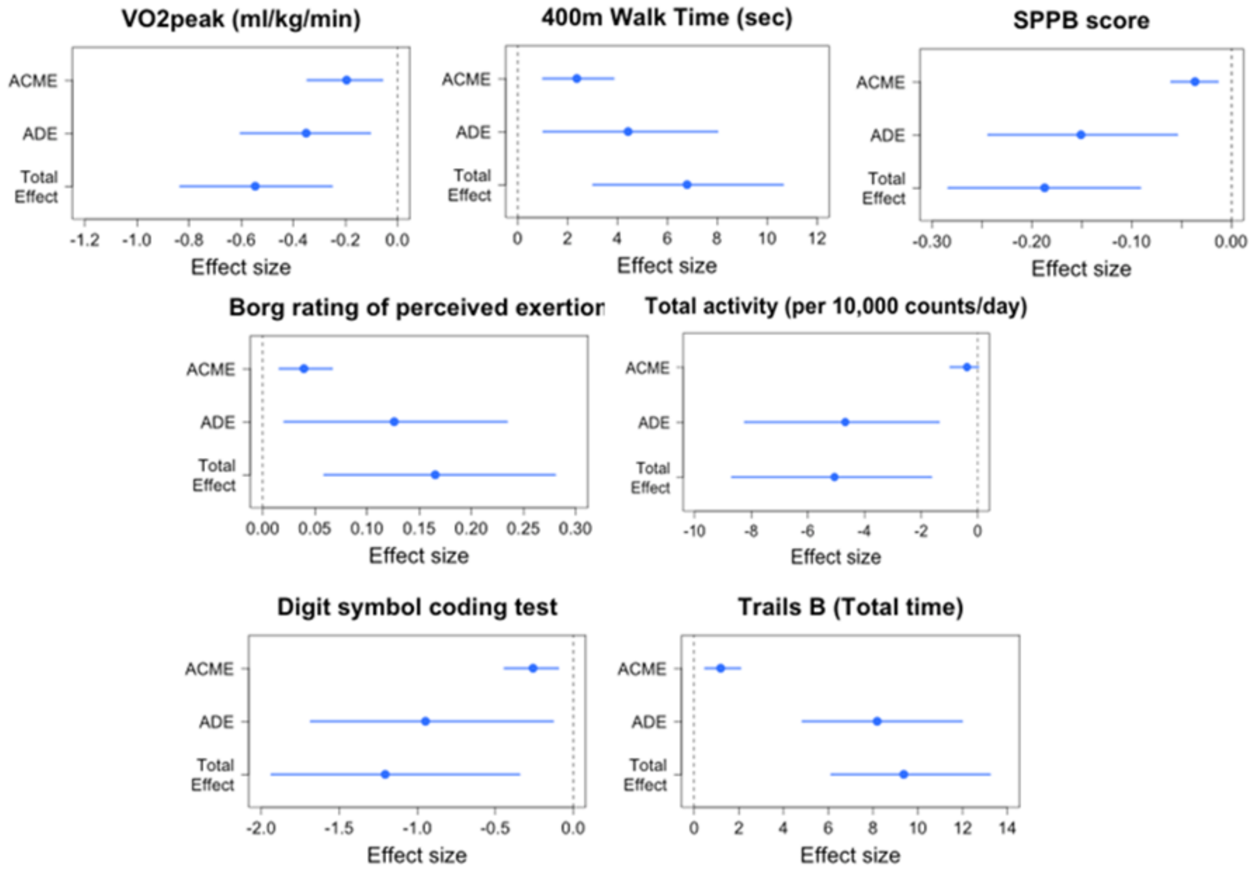

Supplementary Figure 1. Mediation plots of mitochondrial energetics assessed by ATP_{max} in associations between RDW values and diverse physical, and cognitive measurements. ATP_{max} – maximal production of adenosine triphosphate ACME, Average causal mediated effect: ADE, Average direct effect.

© 2025. Kim KM et al. Published online at http://www.aginganddisease.org/EN/10.14336/AD.2024.1724

Supplementary Table 1. Values of RDW and proportions of RDW categories according to physical-, cognitive or both functional impaired groups.

	Normal	Physical function impairment only (SPPB ≤9)	Cognitive function impairment only (Trail B≥180)	Both physical & cognitive functions impairment (SPPB ≤ 9 & Trail B ≥180)	p-value*	ANCOVA p-value (Age & Hgb adjust)
N	548	169	50	58		
RDW (median [IQR]), %	12.8 [12.4;13.2]	12.9 [12.4;13.5]	13.1 [12.5;13.8]	13.2 [12.8;14.0]	<0.001	<0.001
<12	37 (6.8%)	11 (6.5%)	2 (4.0%)	2 (3.4%)	<0.001	
12-12.4	110 (20.1%)	35 (20.7%)	8 (16.0%)	3 (5.2%)		
12.5-12.9	186 (33.9%)	45 (26.6%)	8 (16.0%)	12 (20.7%)		
13-13.4	123 (22.4%)	33 (19.5%)	17 (34.0%)	20 (34.5%)		
13.5-14.4	77 (14.1%)	33 (19.5%)	11 (22.0%)	12 (20.7%)		
≥14.5	15 (2.7%)	12 (7.1%)	4 (8.0%)	9 (15.5%)		

RDW, red cell distribution width; SPPB, Short Physical Performance battery
*P-values were calculated using the Kruskal-Wallis test for continuous variables and the Chi-square test for categorical variables.

© 2025. Kim KM et al. Published online at http://www.aginganddisease.org/EN/10.14336/AD.2024.1724
